# Effects of fluticasone propionate on arachidonic acid metabolites in BAL-fluid and methacholine dose-response curves in non-smoking atopic asthmatics

**DOI:** 10.1155/S0962935196000324

**Published:** 1996-06

**Authors:** S. E. Overbeek, J. M. Bogaard, I. M. Garrelds, F. J. Zijlstra, P. G. H. Mulder, H. C. Hoogsteden

**Affiliations:** 1 Department of pulmonary Diseases University Hospital Rotterdam Dijkzigt and Erasmus University Rotterdam The Netherlands; 2 Department of pharmacology University Hospital Rotterdam Dijkzigt and Erasmus University Rotterdam The Netherlands; 3 Department of Epidemiology and Biostatistics University Hospital Rotterdam Dijkzigt and Erasmus University Rotterdam The Netherlands

## Abstract

Hyperresponsiveness of the airways to nonspecific stimuli is a characteristic feature of asthma. Airway responsiveness is usually characterized in terms of the position and shape of the dose–response curve to methacholine (MDR). In the study we have investigated the influence of fluticasone propionate (FP), a topically active glucocorticoid, on arachidonic acid (AA) metabolites in broncho-alveolar lavage (BAL) fluid (i.e. TxB_2_, PGE_2_, PGD_2_, 6kPGF_1α_ and LTC_4_) on the one hand and MDR curves on the other hand. The effect of FP was studied in a randomized, double-blind, placebo-controlled design in 33 stable nonsmoking asthmatics; 16 patients received FP (500 μg b.i.d.) whereas 17 patients were treated with placebo. We found that the forced expiratory volume in 1s (FEV_1_ % predicted) increased, the log_2_PC_20_ methacholine increased and the plateau value (% fall in FEV_1_) decreased after a 12 week treatment period. No changes in AA-metabolites could be determined after treatment except for PGD_2_ which decreased nearly significantly (p = 0.058) within the FP treated group, whereas the change of PGD_2_ differed significantly (*p* = 0.05) in the FP treated group from placebo. The levels of the other AA metabolites (i.e. TxB_2_, PGE_2_, 6kPGF_1α_ and LTC_4_) remained unchanged after treatment and were not significantly different from the placebo group. Our results support the hypothesis that although FP strongly influences the position, the shape and also the maximum response plateau of the MDR curve, this effect is not mainly achieved by influence on the level of AA metabolites. Other pro-inflammatory factors may be of more importance for the shape of the MDR curve. It is suggested that these pro-inflammatory factors are downregulated by FP.


Mediators of Inflammation, 5, 224–229 (1996)

### Effects of fluticasone propionate on arachidonic acid metabolites in BAL-fluid and methacholine dose–response curves in non-smoking atopic asthmatics

S. E. Overbeek,[1,CA] J. M. Bogaard,[1]
I. M. Garrelds,[2] F. J. Zijlstra,[2] P. G. H. Mulder[3]
and H. C. Hoogsteden[1]

Departments of [1]Pulmonary Diseases, [2]Pharmacology, and [3]Epidemiology and Biostatistics, University Hospital Rotterdam Dijkzigt and Erasmus University, Rotterdam, the Netherlands

[CA]Corresponding Author

**Hyperresponsiveness of the airways to nonspecific stimuli is a characteristic feature of asthma. Airway responsiveness is usually characterized in terms of the position and shape of the dose–response curve to methacholine (MDR). In the study we have investigated the influence of fluticasone propionate (FP), a topically active glucocorticoid, on arachidonic acid (AA) metabolites in broncho-alveolar lavage (BAL) fluid (i.e. $TxB_2$, $PGE_2$, $PGD_2$, $6kPGF_{1\alpha}$ and $LTC_4$) on the one hand and MDR curves on the other hand. The effect of FP was studied in a randomized, double-blind, placebo-controlled design in 33 stable non-smoking asthmatics; 16 patients received FP (500 µg b.i.d.) whereas 17 patients were treated with placebo. We found that the forced expiratory volume in 1 s ($FEV_1$ % predicted) increased, the $\log_2 PC_{20}$ methacholine increased and the plateau value (% fall in $FEV_1$) decreased after a 12 week treatment period. No changes in AA-metabolites could be determined after treatment except for $PGD_2$ which decreased nearly significantly ($p = 0.058$) within the FP treated group, whereas the change of $PGD_2$ differed significantly ($p = 0.05$) in the FP treated group from *placebo*. The levels of the other AA metabolites (i.e. $TxB_2$, $PGE_2$, $6kPGF_{1\alpha}$ and $LTC_4$) remained unchanged after treatment and were not significantly different from the placebo group. Our results support the hypothesis that although FP strongly influences the position, the shape and also the maximum response plateau of the MDR curve, this effect is not mainly achieved by influence on the level of AA metabolites. Other pro-inflammatory factors may be of more importance for the shape of the MDR curve. It is suggested that these pro-inflammatory factors are downregulated by FP.**

**Key words**: Arachidonic acid metabolites, Asthma, BAL fluid, Fluticasone propionate, Glucocorticoids, Methacholine dose–response curve

### Introduction

Bronchial hyperresponsiveness (BHR), a prominent feature of asthma, can be demonstrated by generating dose–response curves through inhalation of histamine or methacholine. Usually these curves are sigmoid in shape, with a distinct threshold, a linear slope in the midpart and a maximum response.[1] The provocative concentration producing a fall of 20% in the $FEV_1$ ($PC_{20}$) is called the sensitivity, whereas the slope in the midpart is defined as reactivity. The plateau value of the curve reflects maximal airway narrowing.

Asthmatics not only show a leftward shift of the dose–response curve, but also higher or even unmeasurable plateau values as compared with normal subjects.[1] Activation of inflammatory cells and release of mediators, such as arachidonic acid (AA) metabolites in bronchoalveolar lavage (BAL) fluid, may be present in asthma and may influence the shape of the methacholine dose–response (MDR) curve by enhancing BHR.[2–5]

Although it is known that anti-inflammatory therapy with inhaled corticosteroids (ICS) shifts the dose–response curve to the right[6] and reduces the maximum response,[7] data concerning the influence of inflammatory mediators such as AA metabolites are scarce.[4] One may expect that inhibition of their release by ICS may lead to a change in the shape of the dose–response curve. Therefore, in asthmatics, we tested the hypothesis that fluticasone propionate (FP), a new topically active ICS, downregulates AA

© 1996 Rapid Science Publishers metabolites in BAL fluid on the one hand, and influences the different characteristics of the MDR curves on the other hand. We also investigated the relation between the various parameters studied.

### Methods

*Subjects:* Thirty-three nonsmoking atopic asthmatics (23 male, median age 26 years, range 18 to 56 years) fulfilled the following criteria: $PC_{20}$ histamine $\leqslant$ 8 mg/ml and $\geqslant$ 9% reversibility in forced expiratory volume in 1 s ($FEV_1$), relative to baseline, following inhalation of 1 000 μg terbutaline sulphate. Atopy was defined by at least one positive skin-prick test to a panel of 16 common aero-allergens in the presence of a positive histamine and negative control.

In the month preceding the run-in period, patients were only allowed to take inhaled short-acting $\beta_2$-agonists, on an as needed basis. All other medication was stopped. Patients with a history suggesting respiratory infection or exacerbation of asthma in the month prior to the study were excluded. All subjects gave informed written consent to the study, which was approved by the local ethics committee.

*Study design:* The study was of a randomized, double-blind, placebo-controlled design. After a run-in period of 2 weeks there was a 12 week treatment period. During the 2 week run-in period patients discontinued use of their usual inhaled bronchodilator which was replaced with salbutamol 400 μg as dry powder via the Diskhaler four times daily. Up to four additional doses were allowed as needed. Baseline data were obtained on two visits with an interval of 2 weeks. At each baseline visit that consisted of two morning or afternoon sessions, a flow volume curve was constructed, bronchodilator response was measured and a provocation test was carried out. At the second baseline visit, intradermal skin testing was performed. When patients fulfilled the mentioned criteria, bronchoalveolar lavage (BAL) was performed 1 week after the last baseline visit. Following the BAL, the patients were randomized to treatment with either inhaled 500 μg FP or placebo, both given twice daily as dry powder via the Diskhaler. Patients continued salbutamol 400 μg four times a day, but could take up to four additional doses as needed for symptomatic relief.

After 6 and 12 weeks of treatment patients attended the clinic on which occasion a MDR curve (see below) was performed. After 13 weeks, 1 week after the last dose–response curve was obtained, the BAL procedure was repeated.

*Long function testing:*

*Inclusion measurements.* $FEV_1$ was derived from a maximal expiratory flow volume curve, using a pneumotachometer (Jaeger, Würzburg, Germany). Reversibility was tested 20 min after four separate inhalations of 250 μg of terbutaline sulphate. A histamine provocation test was performed with a 2 min tidal breathing method[8] using a nose clip. Reference values of lung function measurements are according to Quanjer *et al.*[9]

*Study measurements.* Methacholine was administered according to a standardized tidal breathing method.[10] Dose–response curves were obtained after inhalation of doubling concentrations of acetyl-β-methylcholine bromide (0.03–256 mg $ml^{-1}$ in normal saline). Methacholine and not histamine was chosen as bronchoconstrictor stimulus during the study, because it produces less systemic side effects when given in high doses.[11] Solutions of methacholine were stored at 4°C and administered at room temperature. The aerosols were generated by a De Vilbiss 646 nebulizer (output 0.13 ml $min^{-1}$) and inhaled by tidal breathing for 2 min. The response to methacholine was measured as change in $FEV_1$ expressed as percentage of initial value and related to $\log_2$ dose. A test was interrupted if the $FEV_1$ fell by more than 60%, or if unpleasant side effects or dyspnoea compelled the patient to stop.

A recently developed and validated sigmoid Cumulative Gaussian Distribution (CGD) function was fitted to the data.[12] Although the sensitivity ($\log_2 PC_{20}$) was obtained by linear interpolation of two successive $\log_2$ concentration values,[8] the plateau value and the reactivity (defined as slope in the 50% point of the CGD function) were obtained as best fit parameters. Hence, reactivity denotes the percentual change from baseline $FEV_1$ per doubling dose (%/dd) in the steepest point of the CGD function. Details of the fit procedure and validation of the CGD fit are according to Aerts *et al.*[12]

*Bronchoalveolar lavage:* Fibre-optic bronchoscopy was performed according to guidelines of the American Thoracic Society.[13] After premedication with inhaled terbutaline and atropine i.m., the bronchoscope (Olympus B1 IT 10, Tokyo, Japan) was introduced into the lateral segment of the middle lobe under local anaesthesia and placed in wedge position. BAL was performed with four 50-ml aliquots of sterile phosphate buffered saline (PBS) warmed to 37°C. The fluid was then immediately aspirated by gently suctioning with –40 cm $H_2O$ into a siliconized specimen trap placed on melting ice and transported to the laboratory for processing and analysis.

The BAL fluid was centrifuged at 400 × **g** for 5 min at 4°C. The supernatants were decanted and stored. The cell pellets were then washed in PBS supplemented with 0.5% heat-inactivated bovine serum albumin (BSA). For total leukocyte numbers in BAL fluid, cell suspensions were counted in a Coulter Counter and viability was assessed by cellular exclusion of trypan blue. Cytospin preparations were stained with May–Grünwald–Giemsa stain, and the differential counts were performed by counting at least 500 cells.

*Determination of AA metabolites:* Immediately after the BAL procedure, 20 ml of supernatant was processed on C18 SepPak cartridges (Millipore, Bedford, USA) as described previously,[14] eluated with 2.5 ml methanol and stored at –80°C until analysis. Samples of 200 µl BAL eluted fluid were pipetted into polypropylene tubes and dried with a Savant sample concentrator. After dissolving in 300 µl assay buffer, levels of thromboxane $B_2$ ($TxB_2$) were determined by means of a [$^3$H] RIA with antisera from Advanced Magnetics Inc. (Cambridge, MA) and [$^3$H] labelled compounds from Amersham International (Buckinghamshire, UK). Levels of prostaglandin $PGD_2$ and $PGE_2$ were determined with commercially available [$^3$H] kits (Amersham, UK) and $6kPGF_{1\alpha}$ with a [$^{125}$I] RIA kit (Du Pont de Nemours, Dreieich, Germany), according to the manufacturer's instructions. Leukotriene $C_4/D_4/E_4$ ($LTC_4$) was measured at room temperature in a microtitre enzyme immunoassay according to protocol (Biotrak, Amersham, UK).

*Statistical analysis:* The paired *t*-test was used to analyse within-treatment changes in $FEV_1$ and bronchial hyperresponsiveness. The unpaired *t*-test was used for comparisons between groups. In case of non-normally distributed variables non-parametric tests were used (paired and unpaired Wilcoxon test). A *p*-value smaller than 0.05 was supposed to indicate statistical significance. Spearman–Rank correlations ($r_s$) were used for testing intercorrelation between outcome variables within the groups. Values of $|r_s| > 0.6$ were considered relevant only if they reached a significance level ($p < 0.01$). Group means and standard error of the mean (± S.E.M.) at the various time points were calculated.

### Results

Sixteen patients (12 men) were randomized into the FP group and 17 patients (11 men) into the placebo group. Baseline values such as $FEV_1$, reversibility and $PC_{20}$ histamine were not significantly different between the groups on entry to the study (Table 1). Thirty-one of the 33 subjects completed the study. One patient receiving placebo and one receiving FP were withdrawn after experiencing a pulmonary exacerbation. Data of these two patients have not been included in the analysis.

Mean values for $FEV_1$ (as % predicted), sensitivity ($log_2PC_{20}$methacholine), reactivity (%/dd) and plateau (% fall in $FEV_1$) before and after treatment are shown in Table 2. No statistical significant differences were present between the indices before treatment. In the FP group significant changes occurred after 12 weeks with respect to means of $PC_{20}$ (an increase of 3.6 doubling doses), plateau value (from 58.8% at randomization to 36.5% fall in $FEV_1$) and baseline $FEV_1$ (from 82.9% to 91.0%) in contrast to the placebo group. Changes for reactivity were less marked.

Mean values for AA metabolites and total cell recovery before and after treatment are presented in Table 3. Values were comparable in both treatment groups on entry to the study. Significant results were found only for $PGD_2$; it decreased nearly significantly in the FP group from 25.8 (S.E.M. ± 10.9) to 7.3 (S.E.M. ± 1.8)($p$ = 0.059), whereas its change in the FP group differed significantly from that in the placebo group ($p$ = 0.05) after 12 weeks of treatment.

We also determined if there were correlations

**Table 1.** Baseline characteristics of the study patients

| Characteristics | Fluticasone | Placebo |
|---|---|---|
| *n* | 16 | 17 |
| Sex M/F | 12/4 | 11/6 |
| Age (years) | 28.4 (10.8) | 34.5 (13.5) |
| $FEV_1$ (% predicted) | 84.2 (15.4) | 86.0 (17.6) |
| Reversibility* | 15.5 ( 7.8) | 18.1 (14.3) |
| $PC_{20}H^{\dagger}$ mg/ml | 0.71 (0.59) | 0.88 (0.87) |
| $log_2PC_{20}H^{\dagger}$ | –1.12 (1.62) | –0.96 (1.70) |
| Plateau value‡ | 58.8 (19.8) | 50.8 (12.7) |
| Reactivity (%/doubling dose) | 11.6 ( 5.3) | 9.4 ( 3.7) |
| Total IgE (IU/ml) | 311 (288) | 299 (289) |

All values are expressed as mean ± standard deviation (between brackets).

*Reversibility: change in $FEV_1$ expressed as % baseline, after 1000 µg terbutaline sulphate.

†$PC_{20}H$ = $PC_{20}$ histamine; $log_2PC_{20}H$ - $log_2PC_{20}$ histamine.

‡Plateau value expressed as % fall in $FEV_1$.

No statistical significant differences were present between the treatment groups.

**Table 2.** MDR-curve indices of the study patients

| Lung function parameters | Fluticasone | | Placebo | |
|---|---|---|---|---|
| | Before treatment | After 12 weeks | Before treatment | After 12 weeks |
| $FEV_1$ (% predicted) | 82.9 (4.1) | 91.0 (3.8)*,** | 82.9 (4.2) | 80.1 (5.1) |
| $log_2PC_{20}M^{\dagger}$ | 0.18 (0.56) | 3.77 (0.72)*,** | -0.05 (0.74) | 0.26 (0.46) |
| reactivity (%/doubling dose) | 11.6 (1.3) | 9.0 (1.8) | 9.4 (0.9) | 10.8 (1.0) |
| plateau (% fall in $FEV_1$) | 58.8 (4.9) | 36.5 (4.1)*,** | 50.8 (3.2) | 48.8 (3.6) |

All values are expressed as mean ± S.E.M. (between brackets).
$^{+}log_2PC_{20}M = log_2PC_{20}$ methacholine.
No statistical significant differences were present between the indices before treatment. Significance of changes during treatment are indicated (*$p < 0.05$). Also significant differences in changes between the treatment groups are indicated (** $p < 0.01$).

**Table 3.** AA metabolites and cell levels in BAL fluid

| AA metabolites | Fluticasone | | Placebo | |
|---|---|---|---|---|
| | Before treatment | After 12 weeks | Before treatment | After 12 weeks |
| $TxB_2$ | 34.1 (4.2) | 36.1 (4.2) | 32.2 (4.8) | 29.2 (3.8) |
| $PGE_2$ | 11.3 (0.6) | 10.2 (0.8) | 11.3 (0.6) | 11.3 (0.9) |
| $PGD_2$ | 25.8 (10.9) | 7.3 (1.8) | 15.4 (4.0) | 17.9 (4.5) |
| $6kPGF_{1\alpha}$ | 4.0 (0.4) | 3.2 (0.2) | 4.2 (0.4) | 3.3 (0.5) |
| $LTC_4$ | 12.1 (3.8) | 6.7 (2.0) | 11.6 (4.1) | 12.1 (3.0) |
| Total cell recovery × $10^6$ | 10.8 (0.08) | 15.2 (0.11) | 12.4 (0.09) | 11.4 (0.06) |

All values are expressed as mean ± S.E.M. (between brackets).
AA = arachidonic acid; $TXB_2$ = thromboxane $B_2$; $PGE_2$ = prostaglandin $E_2$; $PGD_2$ = prostaglandin $D_2$; $6kPGF_{1\alpha}$ = 6 keto-prostaglandin $F_{1\alpha}$; $LTC_4$ = leukotriene $C_4$. The decrease in $PGD_2$ within the fluticasone group was nearly significant (p = 0.058). With respect to the difference in changes between both groups only the change in $PGD_2$ differed significantly (p = 0.05) between the fluticasone and the placebo group.

between changes in either treatment group and investigated whether these correlations were different between the treatment groups. In neither of the treatment groups, however, relevant and significant correlations were found between the parameters investigated.

### Discussion

We showed that after 12 weeks of treatment fluticasone propionate (FP) significantly decreased both sensitivity to methacholine and the maximal airway narrowing response, whereas it substantially decreased $PGD_2$ levels in BAL fluid. The change in $PGD_2$ level after treatment with FP was significantly larger than the change in the placebo group. We were unable, however, to demonstrate a correlation between these changes in sensitivity and plateau level with the change in $PGD_2$ or one of the other arachidonic acid (AA) metabolites.

To date, several studies have described the relation between airway inflammation, the subsequent release of AA metabolites and bronchial responsiveness. It has to be kept in mind, however, that the bronchial responsiveness in those studies has never been measured by the entire methacholine dose–response (MDR) curve. Bronchial responsiveness as determined by the MDR curve is defined as the sensitivity of the airways to a wide variety of nonsensitizing bronchoconstricting stimuli. It has been demonstrated that the curves from asthmatics could be differentiatied from those of normal subjects by their position, slope and maximal response.[1] MDR curves in asthma have a steeper slope and a higher maximal response at high doses of methacholine as compared to normal subjects.[1] A leftward shift of the curve can be regarded as being the result of any augmentation of airway narrowing stimuli (i.e. prejunctional mechanisms) such as activation of inflammatory cells and release of mediators such as AA metabolites.[15–17] An upward movement of the plateau is the result of an increase in the response of the effector organ (i.e. postjunctional mechanisms) such as smooth muscle contraction and swelling of the airway wall. To our knowledge this is the first study that investigated the possible correlation of sensitivity, reactivity and plateau value of the MDR curve on the one hand and AA metabolites in BAL fluid on the other hand. However, as mentioned above, we could not demonstrate such a correlation, nor could we find a significant correlation between the FP-induced decrease in BHR and the levels of AA metabolites in BAL fluid. This suggests that in addition to AA metabolites other factors, such as epithelial damage, numbers of eosinophils and neutrophils in BAL fluid, platelet activating factor, histamine and major basic protein, are important as was shown by other authors.[18–22] Our findings are in contrast with those of Oosterhoff *et al.* who demonstrated that the levels of $PGD_2$ in BAL fluid are inversely correlated with the $PC_{20}$ histamine. A possible explanation for this observed discrepancy could be the difference in treatment period; they found a correlation between AA metabolite levels and $PC_{20}$ histamine after long-term treatment (2.5 years) with ICS.

Prostaglandins $D_2$ and $F_{2\alpha}$, thromboxane $B_2$, and leukotrienes $B_4$, $C_4$ and $D_4$ are potent pro-inflammatory mediators with a wide variety of biological activities, including smooth muscle contraction, mucus hypersecretion and leukocyte activation.[23–25] In a previous study we demonstrated that the subclinical inflammation in smokers was associated with higher levels of $PGF_{2\alpha}$ and $TxB_2$ in BAL fluid as compared to non-smokers.[14] It is conceivable that the increased amounts of $PGF_{2\alpha}$ and $TxB_2$ are due to activation of alveolar macrophages in the airways of smokers. $PGF_{2\alpha}$ and $TxB_2$ induce airway secretion, constrict isolated human airways and increase the sensitivity to contractile stimuli.[26] In a study of Wardlaw *et al.* levels of leukotrienes ($LTC_4$ and $LTB_4$) were higher in BAL fluid of symptomatic asthmatics with bronchial hyperresponsiveness as compared to asymptomatics.[27] Although their methodology to measure AA metabolites differed from ours, which makes comparison of the results rather difficult, the levels of $LTC_4$ in our study appeared to be equal to those of the asymptomatics in their study. The fact that these levels were already low at the start of the study may explain why we were unable to demonstrate an effect of treatment.

Other workers carried out allergen challenge and measured BAL fluid and urinary levels of $LTE_4$, the end product of enzymatically converted $LTC_4$ and $LTD_4$. It was found in asthmatics that the basal levels of leukotrienes were not elevated, but increased *in vivo* after allergen challenge.[28–31] Christie *et al.* showed that children with atopic asthma who were resident at high altitude, exhibit a fall in $FEV_1$ and an increase in airway responsiveness to histamine upon visiting regions at sea level. This was associated with a three-fold increase in urinary $LTE_4$ excretion.[32] Thus it seems that in asymptomatic asthma patients or asthmatics with minor symptoms, the levels of leukotrienes in BAL fluid are not increased. Upon stimulation (tobacco smoke, allergen challenge or visit to sea level), however, leukotriene levels rapidly increase resulting in bronchial hyper-responsiveness.

In support of this hypothesis, Bel *et al.* demonstrated that inhaled $LTD_4$ not only caused a higher maximal response plateau than methacholine, but also increased the maximal response to methacholine for at least 3 days. These findings could be prevented by the administration of inhaled steroids.[33]

Tamaoki *et al.*[34] found that prednisone reduced the synthesis of eicosanoids by stimulated macrophage-rich BAL-fluid cells *in vitro.* However, Dworski *et al.* showed that oral prednisone reduced symptoms but had no significant effect on BAL fluid eicosanoid levels *in vivo.*[35] In line with this study, we also failed to find a reduction in AA metabolites in BAL fluid. Only $PGD_2$ appeared to be nearly significantly lower ($p = 0.058$) in FP treated subjects, whereas the change in $PGD_2$ before and after 12 weeks of treatment with FP was significantly larger ($p = 0.05$). It has been demonstrated in canine airway smooth muscle that $PGD_2$ prejunctionally augments the parasympathetic contractile response.[34] Stimulation of asthmatics with $PGD_2$ significantly increased the reactivity to histamine and methacholine.[2] It was suggested that enhanced cholinergic tone underlied these findings. Beasley *et al.* demonstrated that $PGD_2$ and its metabolite $9\alpha,11\beta$-$PGF_2$ caused a marked increase of methacholine induced bronchoconstriction that could only partially be prevented by an anticholinergic.[23] In addition, several studies showed that allergen challenge resulted in a marked increase in prostaglandin levels in BAL fluid.[36,37] This suggests that $PGD_2$ may augment the histamine or methacholine induced hyperresponsiveness. The effects of prostaglandins on reactivity or plateau value is unknown, because none of the above mentioned authors investigated the entire MDR curve and a possible relation of prostaglandins with reactivity or plateau value.

In our study $PGD_2$ levels in BAL fluid of asthmatics substantially decreased after treatment with FP. Since $PGD_2$ is a product derived from mast cells and to a lesser extent from alveolar macrophages,[38,39] it may be concluded from our results that ICS, particularly, downregulate mast cells to produce $PGD_2$ although the influence on alveolar macrophages is not excluded.

In conclusion, in our study we demonstrated that BHR as determined by the MDR curve is downregulated by FP. Although FP strongly influences the position, the shape and also the maximum response plateau of the MDR curve, it did not influence levels of AA metabolites in BAL fluid except for $PGD_2$. Our results indicate that the effect of FP on BHR is not achieved mainly by its influence on the level of the AA metabolites. It is suggested that other pro-inflammatory factors are of more importance for the shape of the MDR curve.

### References

1. Woolcock AJ, Salome CM, Yan K. The shape of the dose–response curve to histamine in asthmatic and normal subjects. *Am Rev Respir Dis* 1984; **130**: 71–75.
2. Fuller RW, Dixon CMS, Dollery CT, Barnes PJ. Prostaglandin $D_2$ potentiates airway responsiveness to histamine and methacholine. *Am Rev Respir Dis* 1986; **133**: 252–254.
3. Leff AR. Endogenous regulation of bronchomotortone. *Am Rev Respir Dis* 1988; **137**: 1198–1216.
4. Bel EH, Van der Veen H, Kramps JA, Dijkman JH, Sterk PJ. Maximal airway narrowing to inhaled leukotriene $D_4$ in normal subjects. Comparison and interaction with methacholine. *Am Rev Respir Dis* 1987; **136**: 979–984.
5. Barnes PJ. Neural control of human airways in health and disease. *Am Rev Respir Dis* 1986; **134**: 1289–1314.
6. Bel EH, Timmers MC, Hermans J, Dijkman JH, Sterk PJ. The long-term effects of nedocromil sodium and beclomethasone dipropionate on bronchial responsiveness to methacholine in non-atopic asthmatic subjects. *Am Rev Respir Dis* 1990; **141**: 21–288.
7. Bel EH, Timmers MC, Dijkman JH, Sterk PJ. The effect of inhaled corticosteroids on the maximal degree of airway narrowing to methacholine in asthmatic subjects. *Am Rev Respir Dis* 1991; **143**: 109–113.
8. Cockcroft DW, Kilian DN, Mellon JJA, Hargreave FE. Bronchial hyperreactivity to inhaled histamine: a method and clinical survey. *Clin Allergy* 1977; **7**: 235–243.
9. Quanjer PH, ed. Standardized lung function testing. *Eur Respir J* 1993; **6**(suppl): 1–95.
10. Hargreave FE, Sterk PJ, Ramsdale EH, Dolovich, J, Zamel N. Inhalation challenge tests and airway responsiveness in man. *Chest* 1985; **87**: 202S–206S.
11. Sterk PJ, Daniel EE, Zamel N, Hargreave FE. Limited bronchoconstriction to methacholine using partial flow–volume curves in nonasthmatic subjects. *Am Rev Respir Dis* 1985; **132**: 272–277.
12. Aerts JGJV, Bogaard JM, Overbeek AFM, Thio P. Extrapolation of methacholine log-dose response curves with a Cumulative Gaussian Distribution function. *Eur Respir J* 1994; **7**: 895–900.
13. Summary and recommendations of a workshop on the investigative use of fiberoptic bronchoscopy and bronchoalveolar lavage in asthmatics. *Am Rev Respir Dis* 1985; **132**: 180–182.
14. Zijlstra FJ, Vincent JE, Mol WM, Hoogsteden HC, Van Hal PThW, Jongejan RC. Eicosanoid levels in bronchoalveolar lavage fluid of young female smokers and nonsmokers. *Eur J Clin Invest* 1992; **22**: 301–306.
15. Sterk PJ, Bel EH. Bronchial hyperresponsiveness: the need for a distinction between hypersensitivity and excessive airway narrowing. *Eur Respir J* 1989; **2**: 267–274.
16. Moreno RH, Hogg JC, Paré PD. Mechanics of airway narrowing. *Am Rev Respir Dis* 1986; **133**: 1171–1180.
17. Fleming W, McPhilips JJ, Westfall DP. Postjunctional supersensitivity and subsensitivity of excitable tissues to drugs. *Rev Physiol Biochem Pharmacol* 1973; **68**: 55–119.
18. Beasly RC, Roberts JA, Roche WR, Holgate ST. Bronchial lavage and biopsy findings before and after allergen challenge in mild atopic asthmatics. *Thorax* 1988; **43**: 812–816.
19. Laitinen LA, Laitinen A. Mucosal inflammation and bronchial hyperreactivity. *Eur Respir J* 1988; **1**: 488–489.
20. Neijens HJ, Degenhart HJ, Raatgreep R, Kerrebijn KF. The correlation between increased reactivity of the bronchi and of mediator-releasing cells in asthma. *Clin Allergy* 1980; **10**: 535–539.
21. Wardlaw AJ, Dunnette S, Cleich G, Collins JV, Kay AB. Eosinophils and mast cells in bronchoalveolar lavage in subjects with mild asthma. Relation to bronchial hyperreactivity. *Am Rev Respir Dis* 1988; **133**: 62–69.
22. Oosterhoff Y, Overbeek SE, Douma R, Noordhoek JA, Postma DS, Hoogsteden HC, Zijlstra FJ. Lower leukotriene $C_4$ levels in bronchoalveolar lavage fluid of asthmatic subjects after 2.5 years of inhaled corticosteroid therapy. *Mediators of Inflammation* 1995; **4**: 426–430.
23. Beasley RCW, Featherstone RL, Church MK, *et al.* Effect of a thromboxane receptor antagonist on $PGD_2$- and allergen-induced bronchoconstriction. *J Appl Physiol* 1989; **66**: 1685–1693.
24. Drazen JM, Austin KF. State of the art: leukotrienes and airway responses. *Am Rev Respir Dis* 1987; **136**: 185–198.
25. Laitinen LA, Laitinen A, Haahtela T, Vikka V, Spur BW, Lee TH. Leukotriene $E_4$ and granulocytic infiltration into asthmatic airways. *Lancet* 1993; **341**: 989–990.
26. De Jongste JC, Mons H, Bonta IL, Kerrebijn KF. *In vitro* responses of airways from an asthmatic patient. *Eur J Respir Dis* 1987; **71**: 23–29.
27. Wardlaw AJ, Hay H, Cromwell O, Collins JV, Kay AB. Leukotrienes, $LTC_4$ and $LTB_4$, in bronchoalveolar lavage in bronchial asthma and other respiratory diseases. *J Allergy Clin Immunol* 1989; **84**: 19–26.
28. Westcott JY, Voelkel NF, Jones K, Wenzel S. Inactivation of leukotriene $C_4$ in the airways and subsequent urinary leukotriene $E_4$ excretion in normal and asthmatic subjects. *Am Rev Respir Dis* 1993; **148**: 1244–1251.
29. O'Shaughnessy KM, Wellings R, Gillies B, Fuller RW. Differential effects of fluticasone propionate on allergen-evoked bronchoconstriction and increased urinary leukotriene $E_4$ excretion. *Am Rev Respir Dis* 1993; **147**: 1472–1476.
30. Taylor GW, Taylor I, Black P, Maltby NH, Turner N, Fuller RW, Dollery CT. Urinary leukotriene $E_4$ after antigen challenge and in acute asthma and allergic rhinitis. *Lancet* 1989; **1**: 584–588.
31. Wenzel SE, Larsen GL, Johnston K, Voelkel NF, Westcott JY. Elevated levels of leukotriene $C_4$ in bronchoalveolar lavage fluid from atopic asthmatics after endobronchial allergen challenge. *Am Rev Respir Dis* 1990; **142**: 112–119.
32. Christie PE, Yntema JL, Tagari P, Ysselstijn H, Ford-Hutchinson AW, Lee TH. Effect of altitude on urinary leukotriene (LT) $E_4$ excretion and airway responsiveness to histamine in children with atopic asthma. *Eur Respir J* 1995; **8**: 837–363.
33. Bel EH, Van der Veen H, Dijkman JH, Sterk PJ. The effect of inhaled budesonide on the maximal degree of airway narrowing to leukotriene $D_4$ and methacholine in normal subjects *in vivo*. *Am Rev Respir Dis* 1989; **139**: 427–431.
34. Tamaoki J, Sekizawa K, Graf PD, Nadel JA. Cholinergic neuromodulation by prostaglandin $D_2$ in canine smooth muscle. *J Appl Physiol* 1987; **63(4)**: 1396–1400.
35. Dworski R, Fitzgerald GA, Oates JA, Sheller JR, Workman R, Prakash C. Effect of oral prednisone on airway inflammatory mediators in atopic asthma. *Am J Respir Crit Care Med* 1994; **149**: 953–959.
36. Liu MC, Hubbard WC, Proud D, *et al.* Immediate and late inflammatory responses to ragweed antigen challenge of the peripheral airways in allergic asthmatics. Cellular, mediator, and permeability changes. *Am Rev Respir Dis* 1991; **144**: 51–58.
37. Wenzel SE, Westcott HY, Smith HR, Larsen GL. Spectrum of prostanoid release after bronchoalveolar allergen challenge in atopic asthmatics and in control groups; an alteration in the ratio of bronchoconstrictive to bronchoprotective mediators. *Am Rev Respir Dis* 1989; **139**: 450–457.
38. Arm JP, Lee T. The pathobiology of bronchial asthma. *Advances in Immunology* 1992; **51**: 323–382.
39. MacDermot J, Kelsey CR, Wadell KA, Richmond R, Knight RK, Cole PJ. Synthesis of leukotriene $B_4$ and prostanoids by human alveolar macrophages: analysis by gas chromatography/mass spectrometry. *Prostaglandins* 1984; **27**: 163–179.

ACKNOWLEDGEMENTS. The authors would like to thank the patients for their enthusiastic participation in the study, the Clinical Research Department of GlaxoWellcome (Drs E. C. G. van Geffen, Dr J. A. M. Raaijmakers), and Professor C. Hilvering, Department of Pulmonology for their continuous support.

**Received 13 March 1996; accepted 3 April 1996**
